# Consensus for a primary care clinical decision-making tool for assessing, diagnosing, and managing shoulder pain in Alberta, Canada

**DOI:** 10.1186/s12875-021-01544-3

**Published:** 2021-10-09

**Authors:** Breda H. F. Eubank, Sebastian W. Lackey, Mel Slomp, Jason R. Werle, Colleen Kuntze, David M. Sheps

**Affiliations:** 1 Department of Health & Physical Education, Faculty of Health, Community, & Education, Mount Royal University, 4825 Mount Royal Gate SW, Calgary, Alberta Canada T3E 6K6; 2 Alberta Bone and Joint Health Institute, Suite 316, 400 Crowfoot Crescent NW, Calgary, Alberta Canada T3G 5H6; 3 Bone and Joint Health Strategic Clinical Network, Alberta Health Services, Seventh Street Plaza, 14th Floor, North Tower, 10030 – 107 Street, Edmonton, Alberta Canada T5J 3E4; 4 Bone and Joint Health Strategic Clinical Network, Alberta Health Services, Seventh Street Plaza, 14th Floor, North Tower, 10030 - 107 Street, Edmonton, Alberta Canada T5J 3E4; 5 Section of Orthopaedic Surgery, Cumming School of Medicine, University of Calgary, 2500 University Drive NW, Calgary, Alberta Canada T2N 1N4; 6 Access Orthopaedics, 3916 Macleod Trail, Suite 300, Calgary, Alberta Canada T2G 2R5; 7 Edmonton Bone and Joint Centre, 9499 - 137 Ave NW, Edmonton, Alberta Canada T5E 5R8; 8 Division of Orthopaedics, Department of Surgery, University of Alberta, 116 St & 85 Ave, Edmonton, Alberta Canada T6G 2R3; 9 Faculty of Rehabilitation Medicine, University of Alberta, 116 St & 85 Ave, Edmonton, Alberta Canada T6G 2R3

**Keywords:** Shoulder pain, Consensus methods, Delphi process, Clinical decision-making, Clinical care pathway

## Abstract

**Background:**

Shoulder pain is a highly prevalent condition and a significant cause of morbidity and functional disability. Current data suggests that many patients presenting with shoulder pain at the primary care level are not receiving high quality care. Primary care decision-making is complex and has the potential to influence the quality of care provided and patient outcomes. The aim of this study was to develop a clinical decision-making tool that standardizes care and minimizes uncertainty in assessment, diagnosis, and management.

**Methods:**

First a rapid review was conducted to identify existing tools and evidence that could support a comprehensive clinical decision-making tool for shoulder pain. Secondly, provincial consensus was established for the assessment, diagnosis, and management of patients presenting to primary care with shoulder pain in Alberta, Canada using a three-step modified Delphi approach. This project was a highly collaborative effort between Alberta Health Services’ Bone and Joint Health Strategic Clinical Network (BJH SCN) and the Alberta Bone and Joint Health Institute (ABJHI).

**Results:**

A clinical decision-making tool for shoulder pain was developed and reached consensus by a province-wide expert panel representing various health disciplines and geographical regions. This tool consists of a clinical examination algorithm for assessing, diagnosis, and managing shoulder pain; recommendations for history-taking and identification of red flags or additional concerns; recommendations for physical examination and neurological screening; recommendations for the differential diagnosis; and care pathways for managing patients presenting with rotator cuff disease, biceps pathology, superior labral tear, adhesive capsulitis, osteoarthritis, and instability.

**Conclusions:**

This clinical decision-making tool will help to standardize care, provide guidance on the diagnosis and management of shoulder pain, and assist in clinical decision-making for primary care providers in both public and private sectors.

**Supplementary Information:**

The online version contains supplementary material available at 10.1186/s12875-021-01544-3.

## Background

Shoulder pain is a highly prevalent condition and a significant cause of morbidity and functional disability [1]. Some patients present with minor symptoms lasting relatively short duration (i.e. less than 3 months) [2]. Other patients present with more severe symptoms lasting long-term (i.e. greater than 12 months), with chronicity and recurrence a common problem [3, 4]. Pain, stiffness, and weakness in the shoulder often lead to chronic pain, disability, and work absenteeism; which affect quality-of-life and burden both patient and society [5, 6]. Shoulder pain also results in financial burden to both the patient and healthcare system. Direct costs include physician services, complementary and allied medical treatments, home healthcare, prescription drugs, hospital inpatient and outpatient costs, ambulatory services, and non-health sector costs [7]. Indirect costs include value of productivity losses due to disability or premature death, and the value of lifetime earnings lost [8].

From a health services perspective, shoulder pain is the second most frequent musculoskeletal (MSK) complaint presenting at the primary care level and the third most common site of MSK pain presenting in the community [9]. Patients presenting with shoulder pain make up one-third of all consultations to primary care physicians [2, 10]. These patients often return for repeat consultations further increasing the burden on public healthcare resources. In Alberta, Canada, over 13,000 patients are seen by primary care physicians for severe shoulder pain [11]. A considerable number of patients also access private healthcare providers for shoulder related treatment, although this magnitude becomes a challenge to qualify due to limitations in information sharing between public and private sectors. Nonetheless, the prevalence of shoulder pain will only continue to increase with the aging population [5].

Patients presenting with shoulder pain require confident assessment, management, and appropriate care pathways. However, current data suggests that many patients presenting with shoulder pain at the primary care level are not receiving such care. Instead the current system is plagued with process inefficiencies, overutilization of diagnostic investigations, inappropriate specialist referrals, and underutilization of appropriate treatment measures; all resulting in lengthy wait times and low quality care [12]. Managing shoulder pain at the primary care level is challenging because many disorders exhibit similar clinical features and lack consensus on diagnostic criteria and concordance in clinical assessment [13]. Unfortunately, primary care physicians often lack the necessary training and confidence to manage shoulder pain appropriately because only 3% of the overall Canadian undergraduate medical curriculum is dedicated to MSK education and they are trained to be generalists [14–16]. Many primary care physicians commonly reach for a prescription pad or request expensive investigations such as magnetic resonance imaging (MRI), which are typically unnecessary and do not provide a clear answer to a clinical question [10]. Additionally, patients presenting to primary care are often referred for specialist care, leading to lengthy wait times for consultations with surgeons and other specialists. This is problematic because most patients do not require surgery yet are referred to surgeons when they could have easily been managed at the primary care level with conservative management, primarily with active, strength-based exercise therapy [12]. Consequently, there is still a level of variation and inappropriateness when treating shoulder pain at the primary care level.

Primary care decision-making is complex and has the potential to influence the quality of care provided and patient outcomes. Patient-centred care requires a structured approach that supports evidence-based decision-making in primary care settings. Therefore, the aim of this project is to develop a clinical decision-making tool to standardize care and minimize uncertainty in assessment, diagnosis, and management for patients presenting to primary care with shoulder pain. The development of this clinical decision-making tool required two stages: 1) identification of evidence-based clinical decision-making tools for shoulder pain; and 2) establishment of provincial consensus for the assessment, diagnosis, and management of patients presenting to primary care with shoulder pain in Alberta, Canada. This includes consensus on diagnostic imaging indications and discriminating between patients that are eligible for surgical and non-surgical treatment options. This project was a highly collaborative effort between Alberta Health Services’ Bone and Joint Health Strategic Clinical Network (BJH SCN) and the Alberta Bone and Joint Health Institute (ABJHI). The BJH SCNs Shoulder Access Project is one of 16 strategic clinical networks established between Alberta’s healthcare system, researchers, and other key partners who have the expertise to improve health outcomes across the province, foster health promotion, and advance prevention work [11]. Several other projects have already demonstrated success in improved system processes such as reduced waiting times, improved efficiency of healthcare resources, and improved patient outcomes for patients presenting with hip and knee joint arthritis, hip fractures, back pain, and acute knee injuries [11, 17–19]. ABJHI is an independent research institute that applies research, data analysis, and measurement by working with healthcare partners such as the BJH SNC to deliver evidence-based, long-term solutions that tangibly improve patient care and patient health. The mandate of both organizations is to transform the healthcare system and ensure that Albertans have access to the right services and providers at the right time, with a focus on innovative service delivery [11].

## Method

### Shoulder Core Design Committee (Project Leadership Group & Advisory Team)

The Shoulder Core Design Committee was formed to guide the development of the clinical decision-making tool for shoulder pain, and consisted of a Project Leadership Group and an Advisory Team. Members of the Project Leadership Group were carefully selected as subject-matter experts. The Project Leadership Group was tasked with managing the project and overseeing a pre-specified, rigorous methodological consensus process. This consisted of a rapid review of the literature, reviewing the evidence, preparing background documents, creating the Delphi questionnaire, analyzing responses, drafting summary reports, and guiding the modified Delphi approach. The Project Leadership Group was comprised of an orthopaedic surgeon, a physical therapist, an athletic therapist, and a quality improvement manager. All 4 members have scientific, clinical, and/or epidemiological backgrounds.

Additionally, an Advisory Team was formed to provide feedback and multidisciplinary guidance to the Project Leadership Group. Members of the Advisory Team were carefully selected because of their previous BJH SCN project experience and included a provincial physical therapist practice lead, a nurse manager, and a patient engagement consultant. The executive director and a senior medical director of the BJH SCN were also consulted.

### Clinical decision-making tool development

#### Rapid review

Initially, the Shoulder Core Design Committee identified six priority areas for the shoulder pain clinical decision-making tool: 1) appropriate clinical presentations to be included in the care pathway; 2) screening criteria to rule out underlying pathologies that require different care pathways; 3) history-taking questions that aided in the differential diagnosis of shoulder pain; 4) physical examination criteria and special tests; 5) benchmark timeline criteria for critical processes, and 6) diagnostic investigation criteria. A rapid review was then conducted to provide up-to-date evidence to support these six priority areas. The rapid review was performed according to the Preferred Reporting Items for Systematic Reviews and Meta-Analyses Protocols (PRISMA-P) guidelines [20]. Within the methodological continuum of assessing evidence, the rapid review method poses a trade-off between time cost and literature-searching scope [21]. Although not as comprehensive as systematic reviews, a rapid review allows for a large body of evidence to be evaluated in a timely manner through a limited, yet structured, literature search [21]. Three electronic databases were searched from inception to June 2019: Medline, EMBASE, and CINAHL. The search strategy was developed and carried out by a health services librarian scientist within the Knowledge Management Department of Alberta Health Services. Articles were limited to English language articles and human studies. The bibliographies of selected articles were also hand searched to identify additional articles not identified by the search strategy. Additional file 1 outlines the search strategy.

Two members of the Project Leadership Group (BE and DS) were responsible for independently evaluating all titles and corresponding abstracts. Prior to the full title and abstract review, both reviewers independently screened a random sample of 50 titles and abstracts (K = 0.66, 95% CI 0.54–0.78). After a full title and abstract review, data was compiled, and consensus was reached for disagreements between the two reviewers regarding potentially relevant articles. Full-text articles of potentially relevant articles were reviewed to determine final study selection using predefined inclusion and exclusion criteria. Articles were included if they identified clinical care pathways, algorithms, clinical practice guidelines, or consensus guidelines that could be used to inform clinical decision-making processes as it related to assessment, diagnosis, management, and treatment of shoulder pain. Additional inclusion and exclusion criteria are presented in Additional file 2.

Data extracted from each article included: authors, publication year, study design, and population. Treatment algorithms (i.e., flowcharts), guidelines, care pathways, appropriate use criteria, and timelines pertaining to management of shoulder pain were also retrieved. Both reviewers independently appraised the body of evidence and provided a grade for each included article based on the Oxford Centre of Evidence-Based Medicine (OCEBM) 2009 model [22]. Randomized clinical trials were considered high-level evidence (level 1), observational studies such as cohort (level 2) and case-control (level 3) studies were considered low-level evidence, and case-series (level 4) and expert opinion (level 5) were considered very-low evidence [22]. This document can be found in Additional file 3. Discrepancies in OCEBM categorization were resolved by discussion until consensus was reached between the two reviewers. The results of the search were used as an evidence-based starting point for the Delphi questionnaire.

#### The modified Delphi approach

The clinical decision-making tool development process utilized a three-round modified Delphi approach to quantify group consensus from March to September 2020. The modified Delphi approach is recommended for use in healthcare settings as a reliable means of determining consensus for a defined clinical problem [23–25]. This method is an iterative process that uses a systematic progression of repeated rounds of voting. It is an effective process for determining expert group consensus where there is little or no definitive evidence and where opinion is important [26]. Initially, a comprehensive list of statements is developed and consensus is built from feedback provided by expert participants from the preceding rounds. The modified Delphi approach deviates from the original Delphi method [27] in that it allows for expert interaction in a final face-to-face meeting. Studies have shown the modified approach to be highly cooperative and effective when developing complex, multi-attribute models [28].

Before rounds of voting began, consensus was defined as ≥80% of experts voting in favour or against a statement (i.e. the summative of “yes” or “no”). Eighty percent was chosen as an appropriate cut off based on work by Lynn [25, 29], who suggested at least 80% expert agreement to achieve content validity when there are at least 10 experts participating in consensus development. The first two rounds of voting were completed via REDCap’s survey distribution tools, which emailed each expert a link to the Delphi questionnaire. Statements that received consensus were removed from the Delphi questionnaire. Statements not meeting consensus were modified according to feedback provided by the expert group. An updated version of the questionnaire was recirculated to the expert group for the next round of voting and also contained group scores and anonymized comments. To maximize responses, experts that had not completed questionnaires within two weeks were sent an email reminder. A final face-to-face meeting had been scheduled for June 2020, however, Covid-19 physical distancing restrictions prevented the meeting from occurring. In lieu, a virtual meeting was held via a web-based platform (Zoom Video Communications, version 5.1.0) in September 2020.

#### Consensus expert selection

Participants for the consensus expert group were purposely selected to represent each stakeholder group and a range of disciplines, expertise, and geographic health regions across Alberta. Experts were chosen to represent family medicine, sports medicine, orthopaedic surgery, radiology, physiatry, athletic therapy, physical therapy, occupational therapy, public policy, and healthcare administration. All experts possessed clinical expertise in assessing and managing shoulder pain. To improve the patient centricity of the tool development process, it was imperative to include a patient advocate [30]. Patient advocates provide a patient’s perspective, which is key to effective care and the development of systems that support that care [31, 32]. Therefore, one patient advocate was selected for the expert group to provide “the patient voice”. The patient previously suffered from shoulder pain and had previous BJH SCN project-oriented research experience.

#### The Delphi questionnaire

The Delphi questionnaire was derived from evidence retrieved from the rapid review. Statements were developed to represent each domain: 11 clinical presentations, 32 screening statements, 29 history-taking statements, 41 physical examination statements, 46 timeline statements, and 112 diagnostic investigation statements. An OCEBM grade was assigned to each statement reflective of the highest level of evidence found. All statements were voted upon using a “yes” or “no” answer format. Respondents were given the option to abstain from voting on statements by selecting “N/A – not applicable to my practice”. The Delphi questionnaire was created in Research Electronic Data Capture (REDCap) software, a secure, web-based software platform designed to support data capture for research studies [33, 34].

#### Clinical scope

This clinical decision-making tool was created with the intention of standardizing assessment, diagnosis, and management for patients presenting with shoulder pain to primary care physicians and allied healthcare professionals in both public and private settings. This tool is suitable for adult men and women (> 18 years old) presenting with shoulder pain resulting from acute or chronic shoulder conditions. Children and young adults (< 18 years old) and patients presenting concomitant symptomatic pathologies (e.g. stroke, multiple sclerosis, inflammatory arthropathy) pose additional concerns that likely require a different standard of care. Therefore, application of this tool for these individuals should be considered with caution.

## Results

### Rapid literature review

**Fig. 1:**
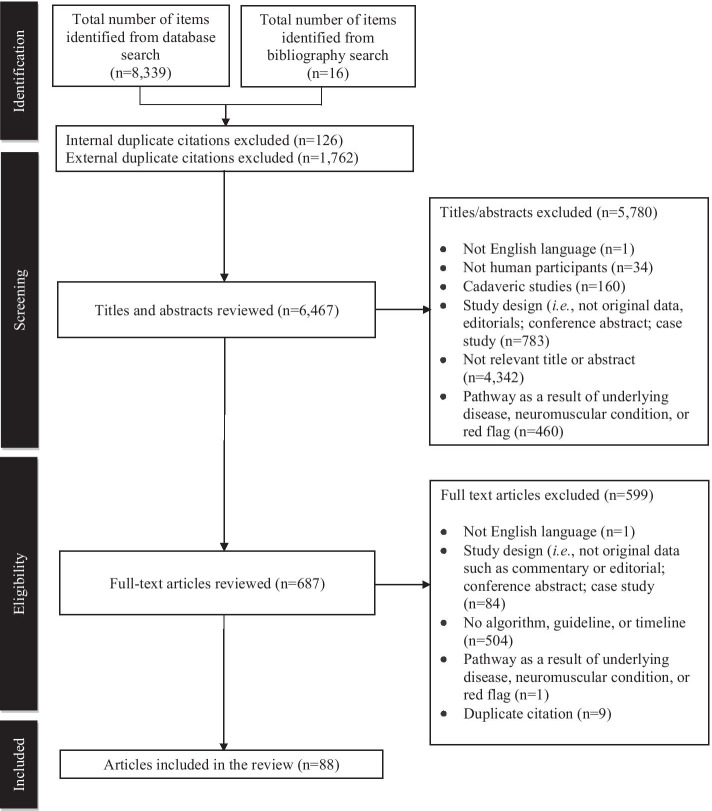
PRISMA-P flow diagram of the identified studies. (Eighty-eight articles were included in the rapid review)

The initial search yielded 8339 articles. A total of 6451 potentially relevant articles were included for review after removing 126 internal and 1762 external duplicates. After applying inclusion and exclusion criteria to the title and corresponding abstracts, 687 articles were selected for full-text review, including 16 articles that were retrieved after searching the bibliographies of selected studies. After a review of full-text articles, 88 articles contained treatment algorithms (i.e., flowcharts), guidelines, care pathways, appropriate use criteria, and timelines pertaining to management of shoulder pain. Although only seven articles were considered high-level evidence, all 88 articles were required to fill in knowledge gaps and used to develop 271 statements representing the best available evidence for the Delphi questionnaire. Figure 1 illustrates the PRISMA-P flow diagram.

### Round 1

**Fig. 2:**
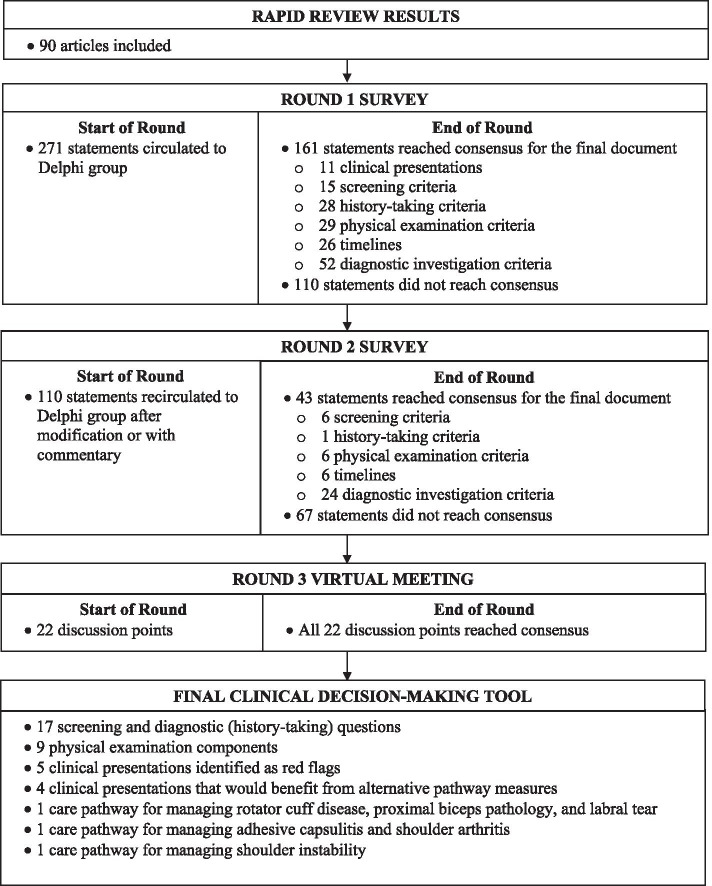
Summary of the Modified Delphi Process

Fifty-five participants were selected for the expert group. The distribution across health disciplines and geographical regions for each round is presented in Table 1. Fifty-one experts completed the Delphi questionnaire in Round 1 resulting in a participation rate of 93%. Statistical analysis allowed the Project Leadership Group to determine the level of consensus achieved for each statement. All 11 clinical presentations reached consensus and were included in the clinical decision-making tool for shoulder pain. Additionally, 15 screening, 28 history-taking, 29 physical examination, 26 timeline, and 52 diagnostic investigation criteria reached consensus. Seventeen screening, one history-taking, 12 physical examination, 20 timeline, and 60 diagnostic investigation criteria did not reach consensus and were retained for voting in Round 2. Figure 2 illustrates the results of the Delphi approach.

**Table 1:** Expert Group Demographic Profile

CATEGORY	BASELINE (***n*** = 55)	Round 1 (***n*** = 51)	Round 2 (***n*** = 46)	Round 3 (***n*** = 30)
**Occupation**				
**Physicians**	**26**	**25**	**23**	**14**
*Orthopaedic surgeon*	*10*	*10*	*9*	*4*
*Sport medicine*	*10*	*9*	*8*	*6*
*Family/General practitioner*	*3*	*3*	*3*	*3*
*Radiologist*	*2*	*2*	*2*	*1*
*Physiatrist*	*1*	*1*	*1*	*0*
**Allied Health**	**28**	**25**	**23**	**15**
*Physiotherapist*	*25*	*24*	*22*	*15*
*Occupational therapist*	*2*	*0*	*0*	*0*
*Athletic therapist*	*1*	*1*	*1*	*0*
**Patient Advocate**	**1**	**1**	0	**1**
**Health Region**				
**Physicians**	**26**	**25**	**23**	**14**
*Calgary*	*7*	*7*	*5*	*4*
*Edmonton*	*11*	*10*	*10*	*7*
*North*	*3*	*3*	*3*	*0*
*Central*	*1*	*1*	*1*	*1*
*South*	*4*	*4*	*4*	*2*
**Allied Health**	**28**	**25**	**23**	**15**
*Calgary*	*14*	*12*	*10*	*8*
*Edmonton*	*7*	*6*	*6*	*5*
*North*	*1*	*1*	*1*	*0*
*Central*	*3*	*3*	*3*	*1*
*South*	*3*	*3*	*3*	*1*
**Patient Advocate**	**1**	**1**	**0**	**1**
*Calgary*	*1*	*1*	*0*	*1*

### Round 2

Only experts that participated in Round 1 were invited to participate in Round 2. Forty-six out of 51 experts completed the Delphi questionnaire resulting in a participation rate of 90%. After Round 2, six screening, one history-taking, six physical examination, six timeline, and 24 diagnostic investigation criteria reached consensus. Eleven screening, six physical examination, 14 timeline, and 36 diagnostic investigation criteria did not reach consensus and were not retained for Round 3 voting.

### Round 3

In all, 204 statements reached consensus from Rounds 1 and 2. Sixty-seven statements failed to reach consensus and were not included in the tool development process. Prior to the virtual meeting, all 204 statements were organized into a visual representation of a draft clinical decision-making tool. The goal was to develop a tool that encompassed a decision-tree approach to assessment, diagnosis, and management of shoulder pain. Gaps within the decision-making process, where evidence was not identified in the rapid review, were filled using the expertise (i.e. best clinical practice) of the Shoulder Core Design Committee. This resulted in 22 new statements that were not previously voted on.

**Fig. 3:**
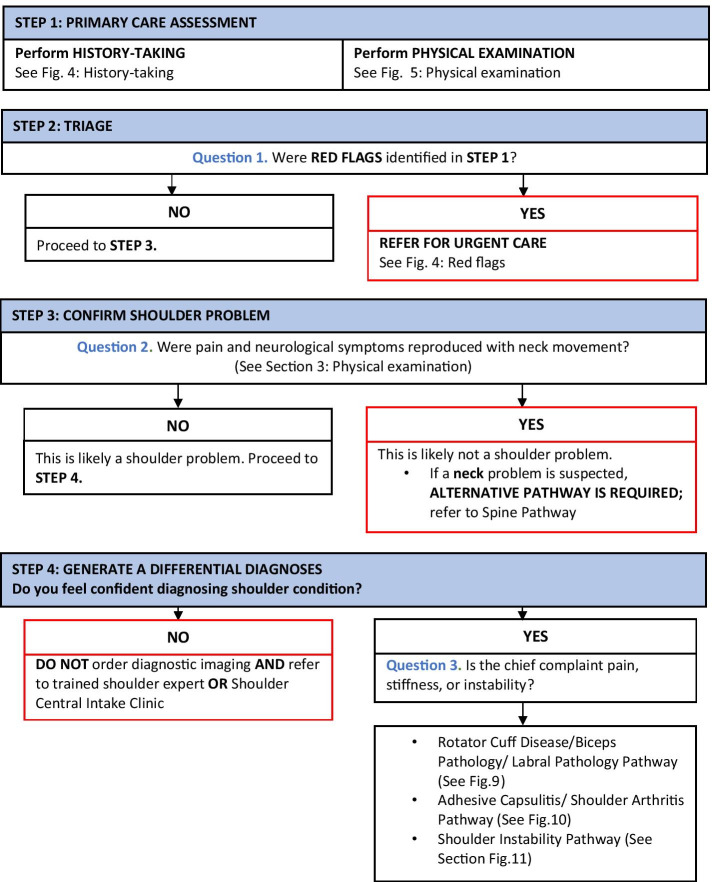
Clinical Management Algorithm for Assessing and Managing Shoulder Pain Presenting to Primary Care

**Fig. 4:**
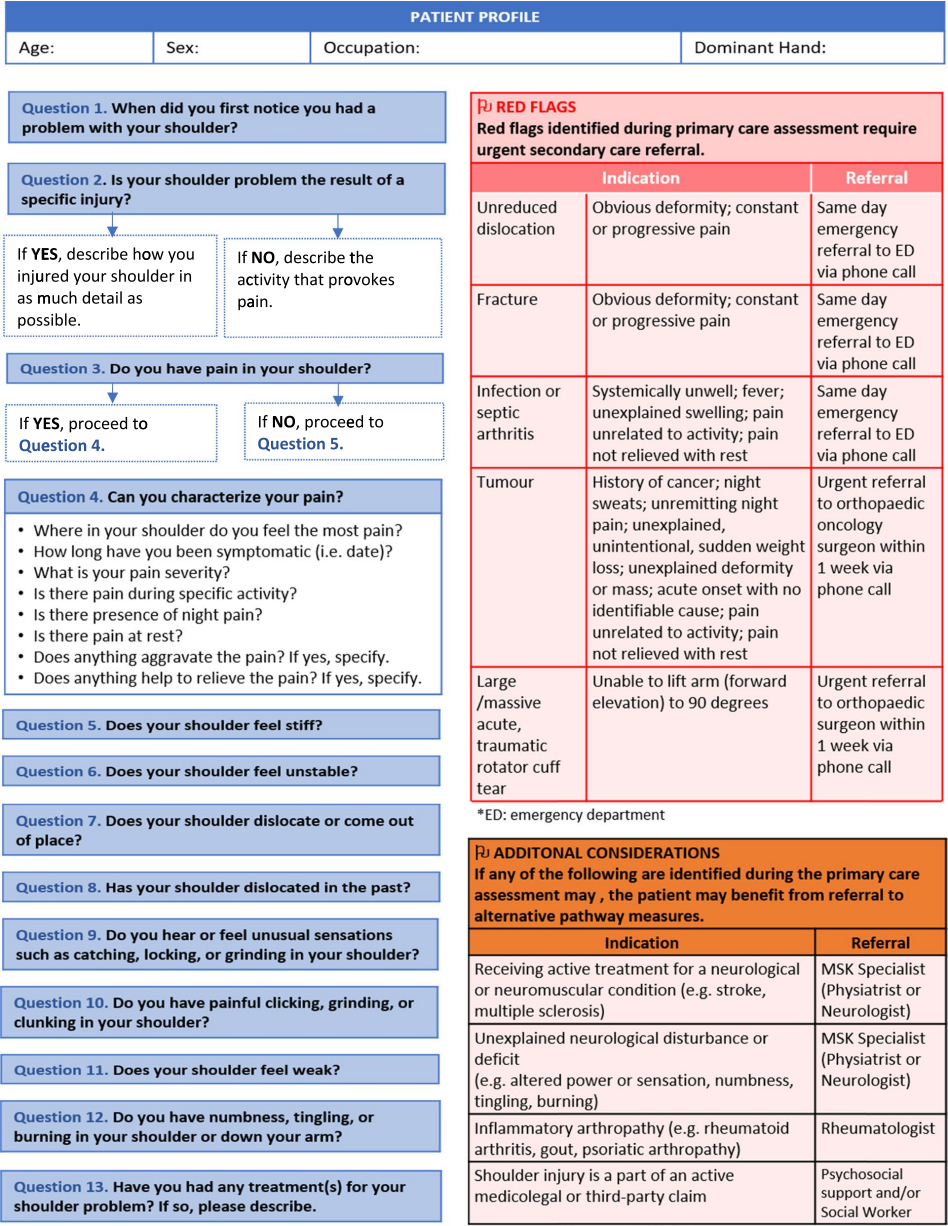
Guide for History-Taking & Identification of Red Flags or Additional Concerns

**Fig. 5:**
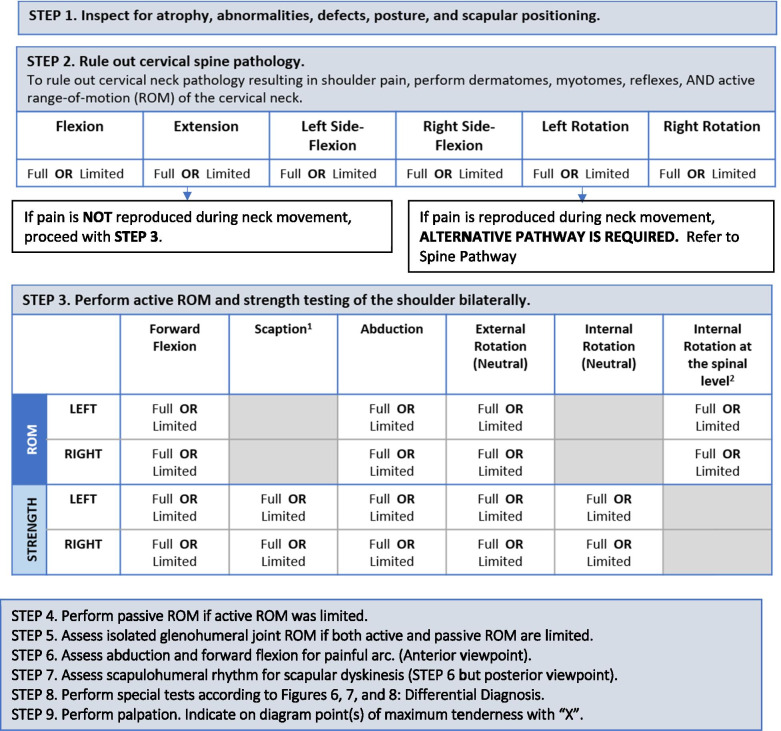
Guide for Physical Examination and Neurological Screening

**Fig. 6:**
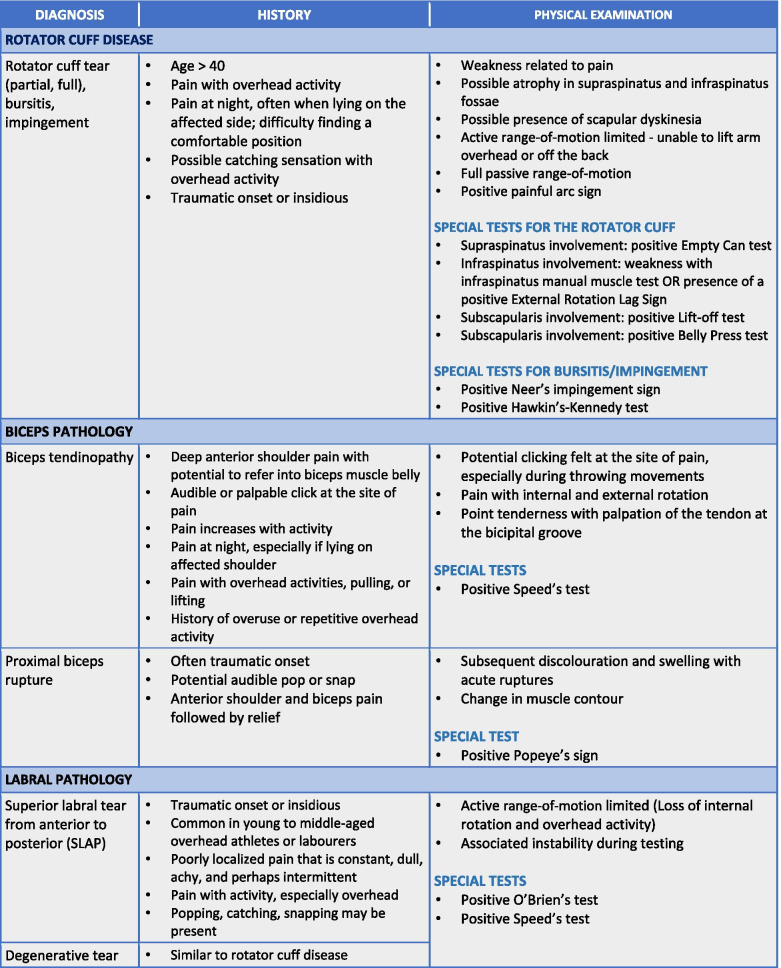
Guide to the Differential Diagnosis of Rotator Cuff Disease, Biceps Pathology & Superior Labral Tear

**Fig. 7:**
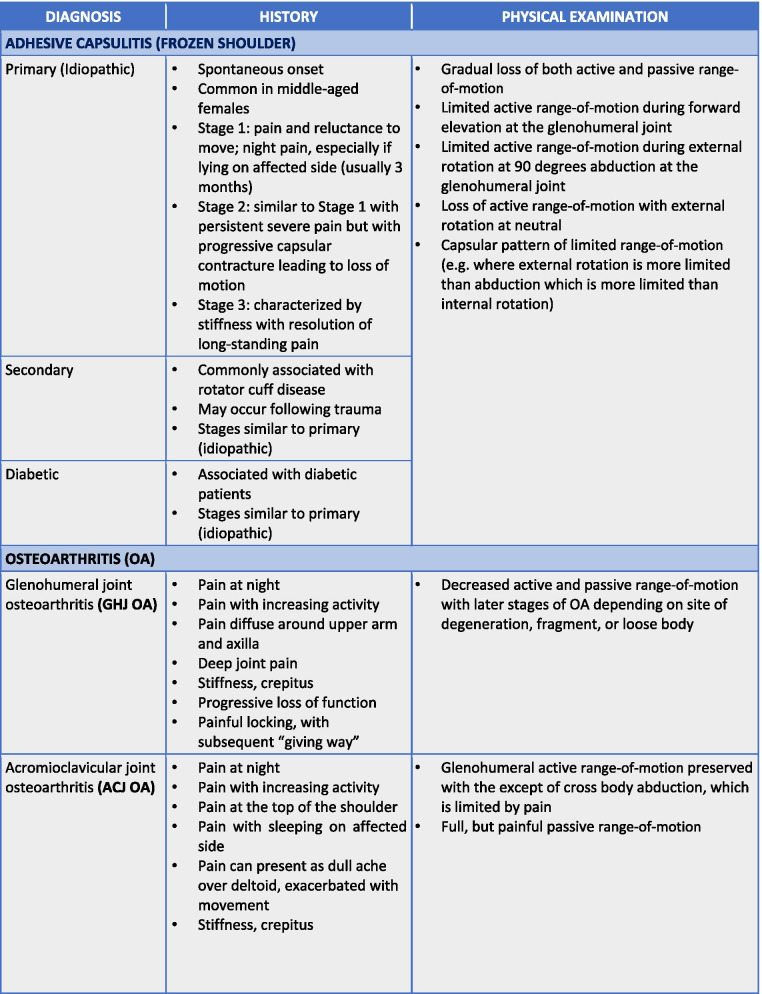
Guide to the Differential Diagnosis of Adhesive Capsulitis and Osteoarthritis

**Fig. 8:**
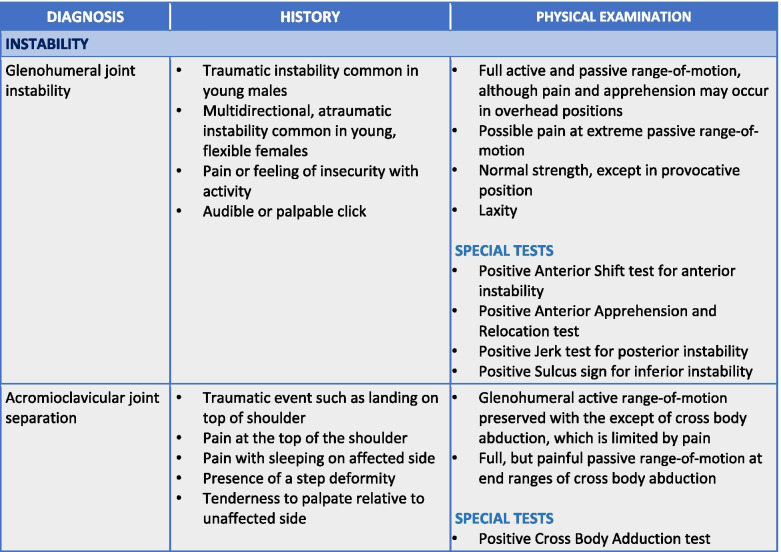
Guide to the Differential Diagnosis of Shoulder Instability

**Fig. 9:**
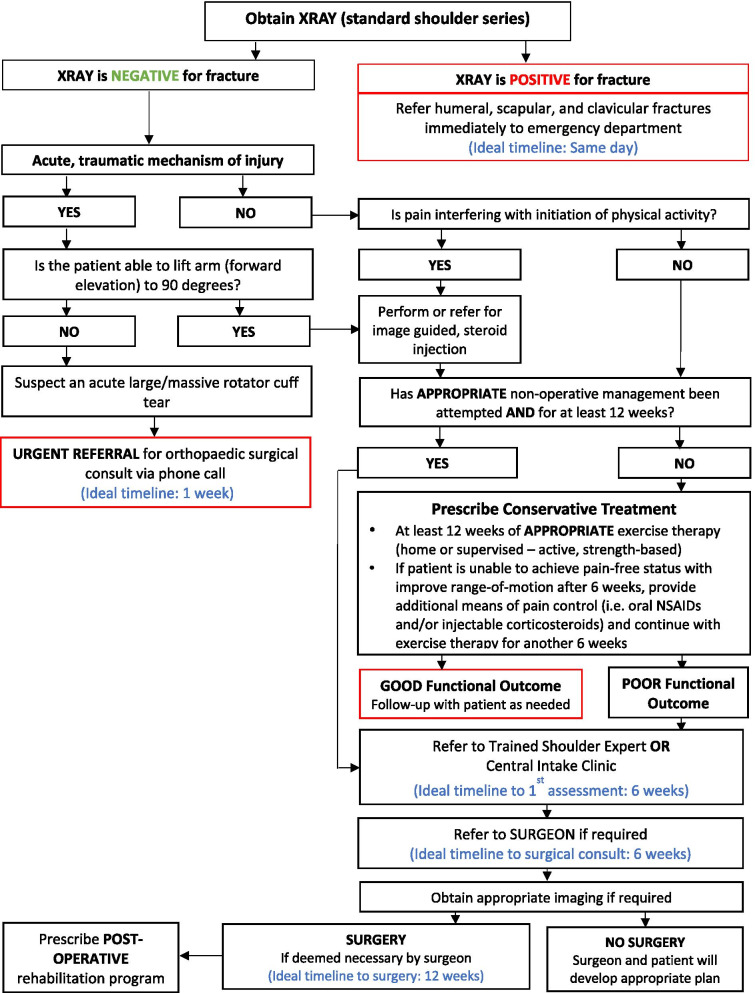
Rotator Cuff Disease, Proximal Biceps Pathology & Labral Tear Care Pathway

**Fig. 10:**
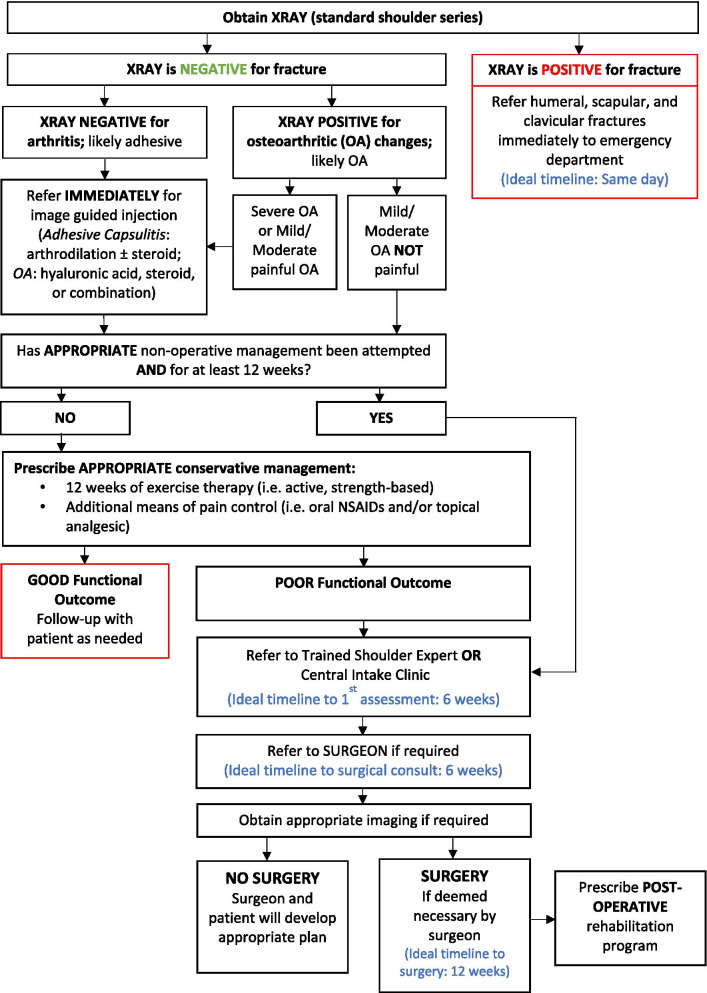
Adhesive Capsulitis & Shoulder Osteoarthritis (OA) Care Pathway

**Fig. 11:**
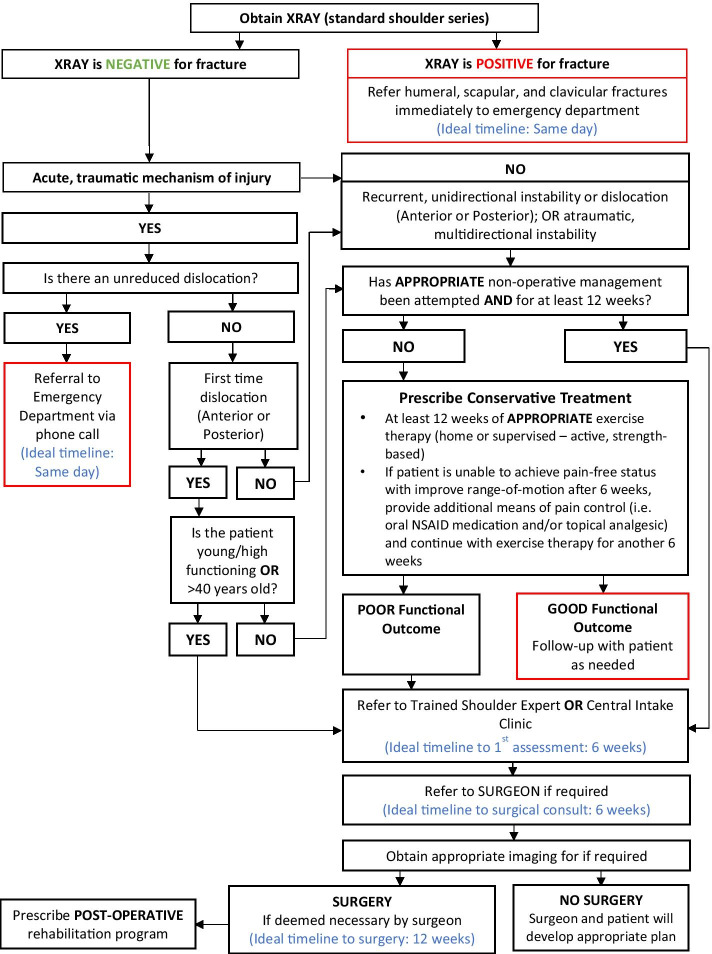
Shoulder Instability Care Pathway

To maximize expert participation for Round 3, the virtual meeting was confined to 2 h. Thirty experts attended the virtual meeting, resulting in a participation rate of 59%. Prior to the meeting, a draft version of the clinical decision-making tool was emailed to the expert group including 22 statements to be discussed during the face-to-face meeting. During the meeting, guided discussions were used to 1) seek consensus for each of the 22 discussion points and 2) each step in the decision-making processes as presented in the draft version of the tool. Discussion and amendments to the tool ensued until consensus was reached for all 22 points including the clinical decision-making tool as a whole. Following the meeting, the tool was updated and recirculated to the entire Delphi group (*n* = 51). Using a Google Ballot Form, experts were asked to confirm their vote for each of the 22 discussion points in addition to agreeing with the tool as presented. Experts were asked to select “agree” or “disagree” for each point on the ballot form. All points met consensus in favour of the final clinical decision-making tool for shoulder pain as presented in Figs. 3, 4, 5, 6, 7, 8, 9, 10 and 11.

### A clinical decision-making tool for shoulder pain

In response to the growing need for consistent standards in assessing, diagnosing, and managing patients presenting with shoulder pain to primary care in Alberta, this clinical decision-making tool for shoulder pain was developed and reached consensus by a province-wide expert panel representing various health disciplines and geographical regions. This tool outlines steps in the clinical decision-making process for primary care providers practicing in both public and private sectors. This tool consists of: a clinical examination algorithm for assessing, diagnosis, and managing shoulder pain (Fig. 3); recommendations for history-taking and identification of red flags or additional concerns (Fig. 4); recommendations for physical examination and neurological screening (Fig. 5); recommendations for the differential diagnosis of rotator cuff disease, biceps pathology, and superior labral tear (Fig. 6); recommendations for the differential diagnosis of adhesive capsulitis and osteoarthritis (Fig. 7); recommendations for the differential diagnosis of shoulder instability (Fig. 8); a care pathway for managing patients with rotator cuff disease, proximal biceps pathology, and labral tears (Fig. 9); a care pathway for managing patients with adhesive capsulitis and shoulder osteoarthritis (Fig. 10); and a care pathway for managing patients with shoulder instability (Fig. 11).

#### Clinical examination algorithm (Fig. 3)

The clinical examination algorithm is a patient-flow diagram consisting of four decision-making steps for managing shoulder pain at the primary care level.

- Step 1 of the algorithm focuses on assessment, which should consist of appropriate history-taking questions (Fig. 4), a physical examination (Fig. 5), and neurological screening (Fig. 5). Figure 4 presents 17 screening and diagnostic questions that reached consensus and should be asked during the history-taking portion of the assessment. Specifically, when obtaining a patient’s medical history, a provider should ask about the patient’s age, sex, occupation, and hand dominance. It is also important to ask about symptoms (pain, stiffness, instability, weakness), pain characteristics, unusual sensations or signs, neurological symptoms (numbness, tingling, burning), past injuries, and past treatments. Figure 5 recommends nine components of the physical examination for shoulder pain. When conducting an appropriate physical examination, it is recommended that the provider should conduct a thorough inspection of the upper torso and shoulders looking for atrophy, abnormalities, and defects. The provider should observe the patient’s posture and scapular positioning as well. A neurological screening exam should be conducted to rule out shoulder pain emanating from cervical spinal pathology. Active and passive range-of-motion, and strength testing should be performed bilaterally. Scapulohumeral rhythm should be assessed for scapular dyskinesis. Figures 6, 7 and 8 outline special tests that should be performed to aid in differential diagnosis. Finally, palpation should be performed to identify the point(s) of maximal tenderness.
- Step 2 of the algorithm highlights the urgency of red flags identified during Step 1 of the primary care assessment. Five clinical presentations were identified as red flags requiring urgent and immediate secondary care referral: unreduced dislocation, fracture, infection or septic arthritis, tumour, and large or massive acute (traumatic) rotator cuff tear. The indications, referral patterns, and benchmark timelines for management are presented in Fig. 4. Four additional clinical presentations were identified, not as red flags, but as additional concerns that would benefit from referral to alternative pathway measures: patients receiving active treatment for a neurological or neuromuscular condition (e.g. stroke, multiple sclerosis); patients with unexplained neurological disturbance or deficit (e.g. altered power or sensation, numbness, tingling, burning); and patients presenting with inflammatory arthropathy (e.g. rheumatoid arthritis, gout, psoriatic arthropathy). It was also recommended that patients presenting with a shoulder injury that is a part of an active medicolegal or third-party claim would benefit from a referral to alternative pathway measures because this population of patients has been found to not improve with standard approaches to treatment [6].
- Step 3 of the algorithm reinforces the need to conduct an appropriate neurological screening exam as part of the physical examination, which is outlined in Fig. 5. The neurological screening exam confirms shoulder pain resulting from acute or chronic shoulder conditions and rules out pain resulting from cervical spinal pathology.
- Step 4 of the algorithm provides the caregiver with two options. First, those that are confident in their primary care assessment and have obtained a differential diagnosis confirmed with the findings presented in Figs. 6, 7, and 8 should proceed down one of three clinical care pathways presented in the tool (Figs. 9, 10 and 11). Alternatively, providers that are not confident in the diagnosis and management of shoulder conditions should not refer patients for diagnostic imaging and instead refer patients to a trained shoulder expert. For example, trained shoulder experts such as non-physician experts are currently employed within team-based models of care in several central access and triage clinics across Alberta. These clinics are designed to manage this population of interest and receive a significant amount of shoulder referrals for their respective geographical region.

#### Diagnostic imaging recommendations

It was recommended that all patients presenting with shoulder pain at the primary care level should be referred for x-ray (i.e. standard shoulder series). If the x-ray is indicative of fracture or unreduced dislocation, the patient requires a same day referral to the emergency department. If the x-ray is negative for acute trauma, providers can proceed down appropriate care pathways presented in Figs. 9, 10, and 11. Additional diagnostic imaging such as MRI is often unnecessary and should not be ordered at the primary care level. MRI requests should be left to the discretion of an orthopaedic surgeon primarily for surgical planning purposes or a trained shoulder expert in collaboration with a specialist.

#### Care pathways

Figure 9 reached consensus as the preferred care pathway for patients suspected of having rotator cuff disease, proximal biceps pathology, or labral tears. If an acute and large or massive rotator cuff is suspected, and the patient is unable to lift their arm to 90^0^ of forward elevation, the patient should be referred immediately such as a phone call to an orthopaedic surgeon within one week of diagnosis. In the absence of a fracture or a large or massive acute (traumatic) rotator cuff tear, it was recommended that an appropriate trial of conservative management be initiated immediately if not already attempted.

Figure 10 reached consensus as the preferred care pathway for patients suspected of having adhesive capsulitis or shoulder osteoarthritis. If an x-ray is positive for osteoarthritic changes and the patient presents with pain, it is recommended that the patient be referred immediately for image-guided injection (i.e. hyaluronic acid, steroid, or combination). If an x-ray is negative for fracture and osteoarthritis, and adhesive capsulitis is suspected, consider referring for image-guided injection (i.e. arthrodilation ± steroid). If an appropriate trial of conservative management has not been attempted, it is recommended that the provider begin a course of action immediately.

Figure 11 is the preferred care pathway for patients suspected of having shoulder instability. An unreduced dislocation was considered a red flag that also requires same day referral to the emergency department. If the patient presents with a first-time reduced dislocation, is high functioning and > 40 years old, the patient should be referred directly to a trained shoulder expert. It is recommended that patients presenting with recurrent, unidirectional or atraumatic, multidirectional instability should begin an appropriate trial of conservative management if not already attempted.

In all three pathways, an appropriate trail of conservative management includes at least 12 weeks of an active, strength-based home or supervised exercise therapy program as the primary treatment option. There is moderate evidence to support additional means of pain control [i.e. oral non-steroidal anti-inflammatory drugs (NSAIDs) and/or injectable corticosteroids] prior to conservative management or at the six week mark if the patient is experiencing difficulty in engaging in exercises due to pain. There is also strong evidence to support manual therapy such as joint mobilizations, manipulations, and soft tissue techniques. Only patients that have failed conservative management should be referred for surgical screening.

## Discussion

The aim of this project was to reach consensus for a clinical decision-making tool with the intent of being adopted by primary care physicians and allied healthcare professionals across Alberta, Canada. This was a 2-stage process that involved a rapid literature review and a modified Delphi consensus approach. The rapid literature review was initially conducted to identify pre-existing clinical decision-making tools that reflected best-practice and evidence-based care. The search failed to identify one comprehensive tool that was inclusive of assessment, differential diagnosis, algorithms, appropriate use criteria for imaging, and benchmark timelines for shoulder pain. However, components of several tools were combined to draft the Delphi questionnaire and initial clinical decision-making tool used during the Delphi approach. The modified Delphi approach was then performed to seek consensus for each statement and to fill gaps in the tool where no literature existed or the evidence was inconclusive. Subsequently, the consensus expert group was able to achieve multidisciplinary consensus on a primary care clinical decision-making tool for shoulder pain. Compared to previous tools identified in the rapid review, robust methodology was used to develop a comprehensive, evidence-based clinical decision-making tool with a strong focus on getting patients to the right provider, in the right order, in the right place, within the right timeframes, and with the right outcomes.

This clinical consensus decision-making tool for shoulder pain is designed to guide patient care in both public and private health sectors, identify red flags, and aid in differential diagnosis. It also provides clarification of provider responsibilities within each step of the care pathway for each of the most common causes of shoulder pain presenting to primary care. The current level of care for patients is plagued with lengthy wait times, variations in quality and access to care, inefficient use of healthcare resources, and lack of coordination between different disciplines and professional specializations [12]. The existence of healthcare silos is another challenge that exists. The ideal model of care would place the patient at the centre, with primary care providers, specialists, public providers, and private providers sharing information and working together to achieve optimal health outcomes. In the current system, there is limited and incomplete information sharing between each silo; thus, resulting in disjointed care as clinicians are not fully aware of previous treatment frequencies, methods, or outcomes. This lack of a patient level record over time restricts the ability to improve the quality of care while impacting patients, providers, and the overall health system. Application and adherence to a clinical decision-making tool would provide a stepwise approach to consistent care, while improving quality and coordination.

The consensus expert group also identified several key factors within the pathway that would improve the current standard of care. First, this clinical decision-making tool highlights the importance of early, conservative management for eligible patients presenting with shoulder pain. The literature suggests that a significant proportion of shoulder pain can be managed successfully with a non-operative approach if the condition is managed early in its course [35–38]. This includes at least 12 weeks of a home or supervised exercise therapy that is focused on active, strength-based exercises with additional means of pain control if required. Therefore, patient education encouraging the adoption of an appropriate exercise therapy plan is recommended to set expectations and promote adherence. In most cases, patients that meet the requirements for conservative management do not need to be seen by a specialist unless the patient has failed treatment with poor functional outcomes. Patients can sometimes wait up to two or more years for an orthopaedic surgical consult [12, 17]. Many wait times exceed the optimal timeframes in providing quality care for patients [12, 17]. A reduction of unnecessary orthopaedic referrals would accurately triage patients to appropriate treatment measures, which would reduce wait lists, save public health care dollars, and improve patient outcomes.

Another key takeaway highlights the importance of reducing inappropriate referral of ancillary tests such as MRI at the primary care level. MRI is an expensive diagnostic test and similar to what has been reported in the literature [25, 30], should be ordered by a surgeon primarily for surgical planning purposes. However, the common misconception at the primary care level is that an MRI is required prior to referral to an orthopaedic surgeon [12, 39]. The average wait time for an MRI in Alberta is 50 weeks [40]. Therefore, treatment becomes delayed and patients spend months on a potentially unnecessary and inappropriate wait list. This not only clogs up the wait list for patients that actually require MRI, but also wastes public health care dollars on unnecessary procedures.

Finally, as part of BJH SCN’s mandate to support evidence-based, innovative ways of delivering high quality healthcare, this clinical decision-making tool highlights the use of central access and triage clinics as a single intake point for shoulder pain referrals. Currently, there are several central access and triage clinics across Alberta that employ a team-based model of care [41]. Within this model, complementary allied health professionals (i.e. athletic therapists, physical therapists) have been trained at a specialist’s competency level and provide a high standard of care as part of an interdisciplinary team with a supervising physician. Similar team-based models of care across Alberta have demonstrated improved quality of care, efficiencies, and patient outcomes with a reduction of publicly funded healthcare services including inappropriate referrals for MRI and specialist physician visits [17, 18, 39, 42]. These clinics provide caregivers that are not confident in diagnosing and managing shoulder pain in Alberta with a simple referral process that streamlines care for eligible patients.

## Strengths and limitations

This project represents the first provincial effort to transform the standard of care for patients presenting with shoulder pain in Alberta. This project standardizes clinical decision-making processes and provides patients and providers a consistent message with respect to evidence-informed, patient-centred care. This project’s success depended on good leadership, broad stakeholder engagement from across the province, establishment of key partnerships, and a commitment to being evidence informed [43]. In line with other BJH SCN projects, clinicians, researchers, patients, and administrators worked collaboratively and remained engaged throughout the entire process.

Originally, expert participants were asked to attend an in-person meeting for Round 3 of the Delphi consensus approach, thus facilitating both small and large group discussion. Although Covid-19 social gathering restrictions prevented an in-person meeting, and rather than wait for a time in which large group gatherings would be allowed, consensus methodology was modified to allow for a virtual meeting. The virtual meeting provided an opportunity for structured group communication and discussions on complex and multi-factorial clinical problems that were too complicated for the original Delphi method. Thus, the virtual meeting allowed the expert group to finalize the contents of the clinical decision-making tool. We believe the shift to a virtual meeting was successful and resulted in a greater amount of expert participation from across the province. One criticism of the modified Delphi approach is the loss of anonymity with the final round of voting; however, the use of a Google Ballot Form allowed for voter anonymity to be maintained.

One criticism of the clinical decision-making tool development process was that some of the literature used did not represent higher grades of evidence. However, a robust, systematic search was utilized to obtain the best available evidence and allowed our experts to translate such evidence into local protocols and clinical pathways. Due to the limited number of high-level evidence identified, failure to incorporate all levels of evidence in the tool development process would have hindered our ability to design and implement a comprehensive patient-centred clinical pathway. Since this tool is a living document, the Shoulder Core Design Committee will continue to update the evidence by engaging key clinical experts and patients to prioritize unanswered questions and fill in evidence gaps. A periodic update, such as a new iteration of the tool, is one anticipated ongoing outcome and deliverable of these activities. Additionally, although the clinical decision-making tool is quite comprehensive, it is not intended for all patients presenting with shoulder pain. Therefore, providers should always consider unique problems not identified by this pathway and have a flexible approach for select patients that present with additional considerations.

## Conclusion

This clinical decision-making tool is intended to provide guidance to primary care physicians and allied health professionals at the primary care level that present with shoulder pain. This tool is to be used in collaboration with the patient as part of the joint decision-making process. It is the opinion of the Shoulder Core Design Committee that this clinical decision-making tool can improve the quality of care for patients with shoulder pain by standardizing a patient’s journey in terms of which treatment should be given, by whom, when, and to what outcome. It represents locally agreed upon, evidence-based, patient-centred best practice care. The implications of this tool for providers outside of Alberta will depend on their local situation. However, this tool can be used for comparison and will aid in the discussion for future improvements. A clinical decision-making tool is relevant to patients, healthcare providers, and policy makers. We believe that this primary care clinical decision-making tool for shoulder pain provides a crucial framework for improving shoulder pain care through evidence synthesis and stakeholder involvement. Next steps should assess the effectiveness and acceptability of this tool. It is the goal of the Shoulder Core Design Committee to revisit and update this pathway every five years.

## Supplementary Information

**Additional file 1.** Search strategies of the literature search

**Additional file 2.** Inclusion and exclusion criteria for rapid literature review

**Additional file 3.** Study characteristics of included rapid review articles

## Data Availability

The dataset supporting the conclusions of this article are available in Scholars Portal Mount Royal University Dataverse (10.5683/SP2/1SWOCI).

## Abbreviations

ABJHI: Alberta Bone and Joint Health Institute
BJH SCN: Bone and Joint Health Strategic Clinical Network
MRI: Magnetic resonance imaging
MSK: Musculoskeletal
MST: Mountain Standard Time
NSAIDs: Non-steroidal anti-inflammatory drugs
OCEBM: Oxford Centre of Evidence-Based Medicine
PRISMA-P: Preferred Reporting Items for Systematic Reviews and Meta-Analyses Protocol
REDCap: Research Electronic Data Capture
