# Supplementary material for: Consensus for a primary care clinical decision-making tool for assessing, diagnosing, and managing shoulder pain in Alberta, Canada
Source: BMC Fam Pract. 2021 Oct 9;22:201. doi: 10.1186/s12875-021-01544-3 (PMC8502088; doi:10.1186/s12875-021-01544-3)
Supplement: Supplementary file 1 — Additional file 1. Search strategies of the literature search [file 12875_2021_1544_MOESM1_ESM.docx]

**APPENDIX A. SEARCH STRATEGIES OF THE LITERATURE SEARCH**

**Search Strategy 1: Ovid MEDLINE(R) and In-Process & Other Non-Indexed Citations and Daily 1946 to June 14, 2019**

| **#** | **Searches** | **Results** |
| --- | --- | --- |
| 1 | shoulder fractures/ or bankart lesions/ | 3208 |
| 2 | Humeral Fractures/ or Shoulder Pain/ | 11732 |
| 3 | shoulder injuries/ or rotator cuff injuries/ or shoulder dislocation/ or shoulder fractures/ or bankart lesions/ or shoulder impingement syndrome/ or bursitis/ | 18656 |
| 4 | (shoulder fracture* or greater tuberosity fracture* or bankart lesion* or bankart fracture* bankart tear* or hill sachs lesion* or shoulder dislocation* or glenohumeral dislocation* or glenohumeral subluxation* or shoulder injur* or rotator cuff injur* or rotator cuff tear* or rotator cuff tendin* or shoulder impingement syndrome* or coracohumeral impingement or posterosuperior glenoid impingement or rotator cuff impingement or frozen shoulder* or adhesive shoulder* or adhesive capsuliti* or bursitis* or shoulder pain or shoulder instabil*).ti. | 9218 |
| 5 | or/1-4 | 31432 |
| 6 | exp Fractures, Bone/ | 176012 |
| 7 | Joint Instability/ | 19163 |
| 8 | Athletic Injuries/ | 26080 |
| 9 | Joint Diseases/ | 24264 |
| 10 | "Sprains and Strains"/ | 5093 |
| 11 | Joint Dislocations/ | 23389 |
| 12 | "wounds and injuries"/ or exp arm injuries/ or soft tissue injuries/ or exp tendon injuries/ | 128293 |
| 13 | arthritis/ or osteoarthritis/ or periarthritis/ | 69678 |
| 14 | (bone fracture* or broken bone* or joint instability* or joint hypermobility* or joint laxit* or athletic injur* or sports injur* or joint diseas* or arthros* or arthropath* or sprain* or strain* or joint dislocation* or injur* or arthriti* or polyarthritis* or osteoarthr* or periarthriti*).ti. | 575991 |
| 15 | or/6-14 | 890254 |
| 16 | Shoulder Joint/ | 18238 |
| 17 | Shoulder/ | 12284 |
| 18 | humerus/ or humeral head/ | 10279 |
| 19 | Clavicle/ | 5605 |
| 20 | scapula/ or acromion/ or coracoid process/ or glenoid cavity/ | 7428 |
| 21 | Acromioclavicular Joint/ | 1962 |
| 22 | Sternoclavicular Joint/ | 1149 |
| 23 | Brachial Plexus/ | 8325 |
| 24 | Rotator Cuff/ | 5903 |
| 25 | Pectoralis Muscles/ | 3750 |
| 26 | biceps.mp. | 12207 |
| 27 | Superficial Back Muscles/ | 820 |
| 28 | triceps.mp. | 9278 |
| 29 | Deltoid Muscle/ | 369 |
| 30 | serratus anterior.mp. | 1204 |
| 31 | rhomboid*.mp. | 2270 |
| 32 | trapezius.mp. | 3867 |
| 33 | (humerus or greater tubercle* or greater tuberosit* or lesser tubercle* or lesser tuberosity* or humeral head* or scapula* or acromion* or coracoid process* or glenoid cavit* or acromioclavicular Joint* or acromioclavicular arch or acromioclavicular ligament* or sternoclavicular Joint* or brachial plexus or rotator cuff* or infraspinatus or subscapularis or supraspinatus or teres minor or pectoral* muscle* or superficial back muscle* or latissimus dors* or trapeziu* or deltoid muscle*).ti. | 28410 |
| 34 | or/16-33 | 94095 |
| 35 | 15 and 34 | 32012 |
| 36 | 5 or 35 | 48794 |
| 37 | critical pathways/ or "delivery of health care"/ or "Continuity of Patient Care"/ | 107952 |
| 38 | guidelines as topic/ or practice guidelines as topic/ | 148116 |
| 39 | "referral and consultation"/ or physician self-referral/ | 63880 |
| 40 | evidence-based practice/ or evidence-based medicine/ or evidence-based emergency medicine/ | 79726 |
| 41 | Clinical Protocols/ | 26701 |
| 42 | Guideline Adherence/ | 30008 |
| 43 | patient care planning/ or case management/ or patient care management/ | 50810 |
| 44 | Models, Theoretical/ | 143897 |
| 45 | algorithms/ | 237633 |
| 46 | consensus/ | 10660 |
| 47 | Clinical Decision-Making/ | 5960 |
| 48 | Practice Patterns, Physicians'/ | 55414 |
| 49 | delphi technique/ | 5126 |
| 50 | (clinical path* or critical path* or clinical care path* or critical care path* or (deliver* adj1 (health care or healthcare)) or community-based or healthcare system* or health care system* or guideline* or refer* or consult* or evidence based or clinical protocol* or treatment protocol* or care plan* or care goal* or case management or model* or algorithm* or consensus or clinical decision making or medical decision making or practice pattern* or prescribing pattern* or delphi).ti. | 823601 |
| 51 | patient care management/ or disease management/ or pain management/ | 67070 |
| 52 | Time-to-Treatment/ | 4904 |
| 53 | decision making, organizational/ or benchmarking/ | 23387 |
| 54 | diagnosis/ or clinical decision-making/ or delayed diagnosis/ or diagnosis, differential/ or diagnostic self evaluation/ | 468839 |
| 55 | (patient care management or disease management or pain management or time to treatment or delayed treatment or organizational decision making or benchmark* or diagnos* or clinical decision making or medical decision making or timeline* or (contin* adj1 patient care)).ti. | 578573 |
| 56 | or/37-55 | 2468577 |
| 57 | 36 and 56 | 5076 |
| 58 | limit 57 to (english language and humans) | 3532 |

**Search Strategy 2: Embase 1974 to 2019 June 14**

| # | Searches | Results |
| --- | --- | --- |
| 1 | exp *shoulder fracture/ | 1084 |
| 2 | exp *humerus fracture/ | 6543 |
| 3 | *shoulder pain/ | 3950 |
| 4 | exp *shoulder disease/ | 20186 |
| 5 | (shoulder fracture* or greater tuberosity fracture* or bankart lesion* or bankart fracture* bankart tear* or hill sachs lesion* or shoulder dislocation* or glenohumeral dislocation* or glenohumeral subluxation* or shoulder injur* or rotator cuff injur* or rotator cuff tear* or rotator cuff tendin* or shoulder impingement syndrome* or coracohumeral impingement or posterosuperior glenoid impingement or rotator cuff impingement or frozen shoulder* or adhesive shoulder* or adhesive capsuliti* or bursitis* or shoulder pain or shoulder instabil*).ti. | 10384 |
| 6 | or/1-5 | 28709 |
| 7 | *fracture/ | 28303 |
| 8 | *joint instability/ | 4325 |
| 9 | *sport injury/ | 17656 |
| 10 | *arthropathy/ | 8792 |
| 11 | *injury/ | 59241 |
| 12 | *joint dislocation/ | 1504 |
| 13 | *arm injury/ | 2878 |
| 14 | *soft tissue injury/ | 2458 |
| 15 | *tendon injury/ | 3987 |
| 16 | *arthritis/ | 26884 |
| 17 | *osteoarthritis/ | 42744 |
| 18 | exp *periarthritis/ | 8000 |
| 19 | (bone fracture* or broken bone* or joint instability* or joint hypermobility* or joint laxit* or athletic injur* or sports injur* or joint diseas* or arthros* or arthropath* or sprain* or strain* or joint dislocation* or injur* or arthriti* or polyarthritis* or osteoarthr* or periarthriti*).ti. | 674568 |
| 20 | or/7-19 | 786762 |
| 21 | *shoulder/ | 9805 |
| 22 | exp *humerus/ | 2835 |
| 23 | exp *clavicle/ | 1772 |
| 24 | exp *scapula/ | 3531 |
| 25 | *acromioclavicular joint/ | 1020 |
| 26 | *sternoclavicular joint/ | 760 |
| 27 | *brachial plexus/ | 3012 |
| 28 | *rotator cuff/ | 2386 |
| 29 | *pectoral muscle/ | 356 |
| 30 | *biceps brachii muscle/ | 1879 |
| 31 | *back muscle/ | 897 |
| 32 | *triceps surae muscle/ or *triceps brachii muscle/ | 962 |
| 33 | *deltoid muscle/ | 581 |
| 34 | serratus anterior.mp. | 1620 |
| 35 | rhomboid*.tw. | 2604 |
| 36 | *trapezius muscle/ | 893 |
| 37 | (humerus or greater tubercle* or greater tuberosit* or lesser tubercle* or lesser tuberosity* or humeral head* or scapula* or acromion* or coracoid process* or glenoid cavit* or acromioclavicular Joint* or acromioclavicular arch or acromioclavicular ligament* or sternoclavicular Joint* or brachial plexus or rotator cuff* or infraspinatus or subscapularis or supraspinatus or teres minor or pectoral* muscle* or superficial back muscle* or latissimus dors* or trapeziu* or deltoid muscle*).ti. | 30691 |
| 38 | or/21-37 | 52587 |
| 39 | 20 and 38 | 10737 |
| 40 | 6 or 39 | 36378 |
| 41 | clinical pathway/ | 8075 |
| 42 | health care delivery/ | 164514 |
| 43 | patient care/ | 276734 |
| 44 | practice guideline/ | 380622 |
| 45 | protocol compliance/ | 10921 |
| 46 | clinical protocol/ | 93429 |
| 47 | patient referral/ | 102097 |
| 48 | evidence based practice/ | 59376 |
| 49 | evidence based medicine/ | 105243 |
| 50 | physician self-referral/ | 766 |
| 51 | theoretical model/ | 81322 |
| 52 | algorithm/ | 244271 |
| 53 | consensus/ | 62060 |
| 54 | clinical decision making/ | 40359 |
| 55 | Delphi study/ | 8578 |
| 56 | (clinical path* or critical path* or clinical care path* or critical care path* or (deliver* adj1 (health care or healthcare)) or community-based or healthcare system* or health care system* or guideline* or refer* or consult* or evidence based or clinical protocol* or treatment protocol* or care plan* or care goal* or case management or model* or algorithm* or consensus or clinical decision making or medical decision making or practice pattern* or prescribing pattern* or delphi).ti. | 1015098 |
| 57 | disease management/ | 17865 |
| 58 | time to treatment/ | 13896 |
| 59 | benchmarking/ | 3508 |
| 60 | diagnosis/ or delayed diagnosis/ or differential diagnosis/ | 1622002 |
| 61 | (patient care management or disease management or pain management or time to treatment or delayed treatment or organizational decision making or benchmark* or diagnos* or clinical decision making or medical decision making or timeline* or (contin* adj1 patient care)).ti. | 665006 |
| 62 | or/43-61 | 4081181 |
| 63 | 40 and 62 | 5513 |
| 64 | limit 63 to (human and english language) | 3623 |

**Search Strategy 3:** **CINAHL**

| **#** | **Searches** | **Results** | |
| --- | --- | --- | --- |
| S1 | (MH "Humeral Fractures+") | 1,863 | |
| S2 | (MH "Shoulder Pain") | 3,488 | |
| S3 | (MH "Shoulder Injuries+") | 7,102 | |
| S4 | TI (shoulder fracture* or greater tuberosity fracture* or bankart lesion* or bankart fracture* or bankart tear* or hill sachs lesion* or shoulder dislocation* or  glenohumeral dislocation* or glenohumeral subluxation* or shoulder injur* or rotator cuff injur* or rotator cuff tear* or rotator cuff tendin* or shoulder impingement syndrome* or coracohumeral impingement or posterosuperior glenoid  impingement or rotator cuff impingement or frozen shoulder* or adhesive shoulder* or adhesive capsuliti* or bursitis* or shoulder pain or shoulder instabil* | 14,024 | |
| S5 | S1 OR S2 OR S3 OR S4 | 16,619 | |
| S6 | (MH "Joint Instability+") | | 8,383 |
| S7 | (MH "Joint Diseases") | 3,971 | |
| S8 | (MH “Wounds and Injuries) OR (MH “Arm Injuries+” OR (MH “Athletic Injuries+) OR (MH “Fractures+”) OR (MH "Soft Tissue Injuries+") OR (MH  "Sprains and Strains+") OR (MH "Tendon Injuries+") | 108,460 | |
| S9 | (MH "Arthritis") OR (MH "Osteoarthritis+") OR (MH "Periarthritis") | 33,195 | |
| S10 | TI bone fracture* or broken bone* or joint instabilit* or joint hypermobility* or joint laxit* or athletic injur* or sports injur* or joint diseas* or arthros* or arthropath* or sprain* or strain* or joint dislocation* or injur* or arthriti* or polyarthritis* or osteoarthr* or periarthriti* | 394,414 | |
| S11 | S6 OR S7 OR S8 OR S9 OR S10 | 429,349 | |
| S12 | (MH "Shoulder Joint+") | 5,181 | |
| S13 | (MH "Shoulder") | 5,789 | |
| S14 | (MH "Clavicle") OR (MH "Humerus") OR (MH "Scapula+") | 4,830 | |
| S15 | MH "Acromioclavicular Joint") OR (MH "Sternoclavicular Joint") | 812 | |
| S16 | (MH "Brachial Plexus+") | 3,939 | |
| S17 | (MH "Rotator Cuff+") OR (MH "Pectoralis Muscles") OR (MH "Biceps Brachii Muscles") OR (MH "Triceps Surae Muscles+") OR (MH "Triceps Brachii") OR  (MH "Deltoid Muscles") OR (MH "Serratus Anterior Muscles") OR (MH "Trapezius Muscles") | 6,145 | |
| S18 | TI humerus or greater tubercle* or greater tuberosit* or lesser tubercle* or lesser tuberosity* or humeral head* or scapula* or acromion* or coracoid process* or glenoid cavit* or articular ligament* or joint capsule* or acromioclavicular Joint*  or acromioclavicular arch or acromioclavicular ligament* or sternoclavicular Joint* or  brachial plexus or rotator cuff* or infraspinatus or subscapularis or supraspinatus or teres minor or pectoral* muscle* or superficial back muscle* or latissimus dors* or  trapeziu* or deltoid muscle* or rhomboid* or superficial back muscle* | 18,818 | |
| S19 | S12 OR S13 OR S14 OR S15 OR S16 OR S17 OR S18 | 30,272 | |
| S20 | S11 AND S19 | 16,215 | |
| S21 | S5 OR S20 | 24,972 | |
| S22 | (MH "Protocols") OR (MH "Critical Path") OR (MH "Patient Care Plans") | 30,166 | |
| S23 | (MH "Health Care Delivery") | 44,580 | |
| S24 | (MH "Practice Guidelines") OR (MH "Guideline Adherence") | 76,598 | |
| S25 | (MH "Referral and Consultation") OR (MH "Professional Practice, Evidence-Based") OR (MH "Medical Practice Evidence-Based") OR (MH "Nursing Practice,  Evidence-Based") OR (MH "Occupational Therapy Practice, Evidence-Based") OR  (MH "Physical Therapy Practice, Evidence-Based") OR (MH "Practice Patterns") | 107,406 | |
| S26 | (MH "Continuity of Patient Care") OR (MH "Disease Management") OR (MH "Pain Management") OR (MH "Case Management") | 48,209 | |
| S27 | (MH "Models, Theoretical") | 40,235 | |
| S28 | (MH "Algorithms") | 31,864 | |
| S29 | (MH "Consensus") OR (MH "Delphi Technique") | 7,240 | |
| S30 | (MH "Decision Making, Clinical") | 27,175 | |
| S31 | TI clinical path* or critical path* or clinical care path* or critical care path* or (deliver* N1 (health care or healthcare)) or community-based or healthcare system* or  health care system* or guideline* or refer* or consult* or evidence based or clinical protocol* or treatment protocol* or care plan* or care goal* or case management or  model* or algorithm* or consensus or clinical decision making or medical decision making or practice pattern* or prescribing pattern* or Delphi | 221,337 | |
| S32 | (MH "Decision Making, Organizational") | 3,217 | |
| S33 | (MH "Benchmarking") | 6,561 | |
| S34 | (MH "Diagnosis") OR(MH "Diagnosis, Differential") OR (MH"Self Diagnosis") OR (MH"Self Assessment") | 82,042 | |
| S35 | TI patient care management or disease management or pain management or time to treatment or delayed treatment or organizational decision making or benchmark* or  diagnos* or clinical decision making or medical decision making or timeline* or (contin* N1 patient care) | 117,657 | |
| S36 | S22 OR S23 OR S24 OR S25 OR S26 OR S27 OR S28 OR S29 OR S30 OR S31 OR S32 OR S33 OR S34 OR S35 | 687,451 | |
| S37 | S21 AND S36 | 3,038 | |
| S38 | S21 AND S36 (Limit English, Limit Human) | 1,184 | |
