# Supplementary material for: Consensus for a primary care clinical decision-making tool for assessing, diagnosing, and managing shoulder pain in Alberta, Canada
Source: BMC Fam Pract. 2021 Oct 9;22:201. doi: 10.1186/s12875-021-01544-3 (PMC8502088; doi:10.1186/s12875-021-01544-3)
Supplement: Supplementary file 2 — Additional file 2. Inclusion and exclusion criteria for rapid literature review [file 12875_2021_1544_MOESM2_ESM.docx]

**Appendix A. Inclusion and exclusion criteria for rapid literature review**

| **Inclusion Criteria** | **Exclusion Criteria** |
| --- | --- |
| 1. Original data 2. Study design (ideally prospective cohort and interventional only however depending on numbers we may need to include retrospective and/or cross sectional – this will be decided after full text retrieval) 3. Consensus conference proceedings or consensus methods 4. Reviews, systematic reviews, and meta-analyses 5. Shoulder pathology to include acute, chronic, and acute-on-chronic shoulder injuries.  - Acute shoulder injuries as defined by a discrete traumatic episode resulting in an injury to a previously asymptomatic shoulder. - Chronic shoulder injuries as defined by shoulder pain of insidious or gradual onset or resulted from a previous traumatic episode. - Acute-on-chronic shoulder injuries as defined by a pre-existing rotator cuff pathology who experiences a traumatic episode to the ipsilateral shoulder.  1. Shoulder pathology to include the following:  - Acromioclavicular joint injury (acute, chronic, acute-on-chronic) - Adhesive capsulitis - Arthritis (periarthritis, osteoarthritis) - Brachial plexus injury and/or other nerve pathology - Bursitis - Fractures (humerus, clavicle, scapula) - Glenohumeral joint instability (acute, chronic, acute-on-chronic) - Glenoid labrum injury - Other muscle injuries (muscle strains – biceps group, triceps group, deltoid group, rotator cuff, pectoralis major/minor, serratus anterior, rhomboids group, trapezius) - Other tendon injuries (distal pectoralis major rupture, proximal biceps tendon rupture, latissimus dorsi rupture of humeral attachment) - Rotator cuff pathology (partial-thickness tear, full-thickness tear, impingement) - Sternoclavicular joint injury (acute, chronic, acute-on-chronic) | 1. Not human subjects 2. Cadaveric studies 3. Study design (no original data, conference abstract only, case study, or case series with fewer than 10 patients) 4. Not written in the English language 5. Tool evaluation and measurements 6. Therapeutic modalities 7. Surgical techniques 8. Physical examination or special tests 9. Not pain referred from substantial cervical spine pathology 10. Not pain as a result of chronic pain syndrome (myofibralgia, etc.) 11. Not pain as a result of underlying disease or neurological or neuromuscular condition such as  - stroke - multiple sclerosis - diabetes - ischemic heart disease - renal disease - respiratory disease - cancer - arthritis involving multiple joints  1. Not red flags such as:  - cancer/tumour - infection - auto-immune disease - inflammatory arthritis |
