# Supplementary material for: Consensus for a primary care clinical decision-making tool for assessing, diagnosing, and managing shoulder pain in Alberta, Canada
Source: BMC Fam Pract. 2021 Oct 9;22:201. doi: 10.1186/s12875-021-01544-3 (PMC8502088; doi:10.1186/s12875-021-01544-3)
Supplement: Supplementary file 3 — Additional file 3. Study characteristics of included rapid review articles [file 12875_2021_1544_MOESM3_ESM.docx]

**APPENDIX B. Study Characteristics of Included Rapid Review Articles**

|  | **Authors and Publication Year** | **Shoulder Pathology** | **Study Design** | **Highest Level of Evidence** | **Findings** |
| --- | --- | --- | --- | --- | --- |
| 1 | Reid et al., 2012 | Acromioclavicular joint (ACJ) separations | Systematic review | 3a | Developed an algorithm for rehabilitation of ACJ separation injury grades I-III. |
| 2 | Boselli et al., 2010 | Glenohumeral (GH) arthrosis | Literature review | 5 | Developed an algorithm for the management of focal chondral lesions as a result of glenohumeral arthrosis. |
| 3 | Izquierdo et al., 2010 | Glenohumeral (GH) osteoarthritis | Systematic review/consensus guidelines | 2a | Established clinical practice guidelines for the treatment of GH osteoarthritis. |
| 4 | Robinson et al., 2012 | Adhesive capsulitis | Literature review | 5 | Developed two algorithms: one for the investigation and treatment of adhesive capsulitis and the second for the treatment for adhesive capsulitis. |
| 5 | Lamplot et al., 2018 | Adhesive capsulitis | Cohort | 3b | Developed an algorithm for treating adhesive capsulitis. |
| 6 | Kelley et al., 2013 | Adhesive capsulitis | Systematic review | 2a | Developed clinical practice guidelines and an algorithm for the treatment of adhesive capsulitis. |
| 7 | Lewis, 2015 | Adhesive capsulitis | Literature review | 5 | Developed descriptive care pathway for patients with adhesive capsulitis. |
| 8 | Mitra et al., 2009 | Adhesive capsulitis | Retrospective cohort | 2b | Developed an algorithm for treating patients to improve passive range of motion. |
| 9 | Morrissey, 2006 | Adhesive capsulitis | Qualitative study | 5 | Developed an algorithm for treating patients with adhesive capsulitis. |
| 10 | Rotini et al., 2005 | Adhesive capsulitis | Retrospective chart review | 4 | Developed an algorithm for treating patients with adhesive capsulitis. |
| 11 | Thomas et al., 2011 | Adhesive capsulitis | Retrospective cohort | 2b | Developed an algorithm for treating patients with manipulation under anaesthesia. |
| 12 | Ogon et al., 2009 | Adhesive capsulitis | Prospective cohort | 1b | Established ideal timelines for treatment of adhesive capsulitis. |
| 13 | Martin et al., 2019 | Brachial plexus injuries | Systematic review | 2a | Established ideal timelines for operating on traumatic stretch and blunt brachial plexus injuries. |
| 14 | McGillicuddy, 1991 | Brachial plexus injuries | Literature review | 5 | Developed an algorithm for the evaluation and management of brachial plexus injuries. |
| 15 | Pondaag et al., 2019 | Brachial plexus injuries | Retrospective chart review | 4 | Developed an algorithm to identify candidates eligible for early surgery following brachial plexus traction injury as a result of high kinetic trauma. |
| 16 | Sinha et al., 2016 | Brachial plexus injuries | Literature review | 5 | Developed six algorithms: two for the management of brachial plexus injuries (closed and open injuries) and four surgical strategies directed towards restoration of nerve function (C5/C6, C5-C7, C8-T1, and C5-T1). |
| 17 | Yoshikawa et al., 2006 | Brachial plexus injuries | Literature review | 5 | Developed imaging guidelines: recommended CT myelography as the initial imaging modality, preferably with the addition of standard myelography and contrast-enhanced magnetic resonance imaging. |
| 18 | Allen & Dean, 2002 | Axillary nerve dysfunction | Expert opinion | 5 | Developed an algorithm for the management of axillary nerve dysfunction. |
| 19 | Avis & Power, 2018 | Axillary nerve injury associated with GH dislocation | Literature review | 5 | Developed two algorithms: one for the detection of high grade axillary nerve injury and one for the management of axillary nerve injury. |
| 20 | Chen et al., 2016 | Acute isolated axillary nerve injury | Retrospective chart review | 4 | Developed a surgical algorithm for acute isolated axillary nerve injuries. |
| 21 | Steinmann & Moran, 2001 | Isolated axillary nerve injury | Literature review | 5 | Developed an algorithm for acute and chronic isolated axillary nerve lesions. |
| 22 | Pandya et al., 2010 | Humeral fractures in the pediatric population | Retrospective chart review | 4 | Established three predictors of abuse for patients presenting with humerus fractures: <18 months old, history (suspicious for abuse), and physical and/or radiographic evidence of prior injury. |
| 23 | Anderson, 2003 | Distal type II clavicle fracture | Literature review | 5 | Developed an algorithm for the treatment of type II distal clavicle fractures based on initial fracture displacement. |
| 24 | Stepanyan et al. 2017 | Simple clavicle fractures in adolescents | Prospective cohort | 1b | Developed an algorithm for the management of clavicle fractures. |
| 25 | Kokkalis et al., 2017 | Posterior shoulder fracture-dislocations | Literature review | 5 | Developed an algorithm for managing patients with posterior shoulder fracture-dislocations. |
| 26 | Kancherla et al., 2017 | Proximal humeral fracture | Literature review | 5 | Developed a surgical algorithm for managing proximal humerus fractures. |
| 27 | Kaya et al., 2017 | Proximal humeral fracture | Literature review | 5 | Developed an algorithm for managing proximal humerus fractures. |
| 28 | Misra et al., 2019 | Proximal humeral fracture | Literature review | 5 | Developed an algorithm for managing proximal humerus fractures. |
| 29 | Pinkas et al., 2014 | Malunion of proximal humeral fracture | Literature review | 5 | Developed three surgical algorithms for managing malunion fractures of the proximal humerus. |
| 30 | Siegel & Dines., 2004 | Malunion of proximal humeral fracture | Literature review | 5 | Developed an algorithm for managing 2-part surgical neck malunion. |
| 31 | Spross et al., 2019 | Proximal humeral fracture | Prospective cohort | 1b | Developed two algorithms for managing proximal humerus fractures (< 65 years and ≥ 65 years). |
| 32 | Hawkins et al., 1986 | Anterior GH dislocation | Prospective cohort | 1b | Established three recommendations for managing anterior shoulder dislocations. |
| 33 | Lin et al., 2018 | Anterior GH dislocation | Literature review | 5 | Developed an algorithm for first-time traumatic anterior shoulder dislocations in pediatric and adolescent patients. |
| 34 | Boone & Arciero, 2010 | Anterior GH dislocation | Literature review | 5 | Developed algorithm for treating first-time anterior shoulder dislocations. |
| 35 | Emond et al., 2009 | Anterior GH dislocation | Prospective cohort | 1b | Developed an algorithm that outlines a clinical decision rule to guide pre- and postreduction radiography for the emergency department for patients with anterior shoulder dislocation. |
| 36 | Hutyra et al., 2019 | Anterior GH dislocation | Retrospective chart review | 4 | Developed an algorithm that outlines the clinical care pathway for managing patients presenting with first-time anterior shoulder dislocations. |
| 37 | Lewis et al., 2016 | Posterior GH dislocation | Literature review | 5 | Developed algorithm for treating posterior shoulder dislocations. |
| 38 | Paul et al., 2011 | Posterior GH dislocation | Systematic review | 2a | Developed algorithm for treating posterior shoulder dislocations. |
| 39 | Bak et al., 2010 | GH instability | Consensus methods | 5 | Established a consensus statement on shoulder instability with respect to evaluation (physical examination, classification, soft-tissue evaluation), treatment (nonoperative and surgical), and patient-reported outcome measures. |
| 40 | Burgess & Sennett, 2003 | GH instability | Literature review | 5 | Developed algorithm for treating traumatic shoulder instability. |
| 41 | Cody & Strickland, 2014 | GH instability | Systematic review | 2a | Developed a surgical algorithm for treatment of multidirectional instability in female athletes. |
| 42 | Paxton et al., 2014 | GH instability | Literature review | 5 | Developed algorithm for treating posterior shoulder instability in older patients. |
| 43 | Spiegl et al., 2013 | Labrum - Bankart | Retrospective chart review | 4 | Developed an algorithm for treating acute traumatic osseous Bankart lesions resulting from first time shoulder dislocations.  |
| 44 | Brockmeyer et al., 2016 | Labrum - SLAP | Literature review | 5 | Classified tears of the superior labrum anterior-to-posterior (SLAP) lesions and developed a treatment algorithm. |
| 45 | Werner et al., 2014 | Labrum - SLAP | Literature review | 5 | Developed two algorithms for treatment of failed SLAP repairs. |
| 46 | Khazzam et al., 2012 | Long head of biceps tendinopathy | Literature Review | 5 | Developed an algorithm for the diagnosis and management of long head of biceps tendinopathy. |
| 47 | Aboelmagd et al., 2018 | Rotator cuff | Literature Review | 5 | Developed an algorithm for treating rotator cuff pathology. |
| 48 | Arce et al., 2013 | Rotator cuff | Consensus methods | 5 | Compiled consensus document for the treatment and management of rotator cuff tears. |
| 49 | Aumiller & Kleuser, 2015 | Rotator cuff | Literature Review | 5 | Developed an algorithm for treating rotator cuff pathology. |
| 50 | Bartolozzi et al., 1994 | Rotator cuff | Prospective cohort | 1b | Developed an algorithm for treating rotator cuff pathology. |
| 51 | Beaudreuil et al., 2010 | Rotator cuff | Systematic review/consensus guidelines | 2a | Established clinical practice guidelines for indications for surgery and surgical techniques. |
| 52 | Brier, 1992 | Rotator cuff | Literature Review | 5 | Developed an algorithm for treating rotator cuff pathology. |
| 53 | Dines et al., 2006 | Rotator cuff | Literature Review | 5 | Developed an algorithm for treating irreparable rotator cuff tears. |
| 54 | Duncan et al., 2015 | Rotator cuff | Prospective cohort | 1b | Established timeline for surgery for repair of acute rotator cuff tears. |
| 55 | Edwards et al., 2016 | Rotator cuff | Literature Review | 5 | Developed an algorithm for treating rotator cuff pathology. |
| 56 | Eubank et al., 2016 | Rotator cuff | Systematic review/consensus guidelines | 2a | Developed two algorithms for treating rotator cuff pathology: acute & chronic/acute-on-chronic. |
| 57 | Frieman et al., 1994 | Rotator cuff | Literature Review | 5 | Developed an algorithm for treating rotator cuff pathology. |
| 58 | George & Khazzam, 2012 | Rotator cuff | Literature Review | 5 | Developed an algorithm for revision rotator cuff repair for symptomatic failed rotator cuff repair. |
| 59 | Greenspoon et al., 2015 | Rotator cuff | Literature Review | 5 | Developed an algorithm for treating massive rotator cuff tears. |
| 60 | Guckel & Nidecker, 1997 | Rotator cuff | Literature Review | 5 | Developed an algorithm for diagnostic imaging of rotator cuff pathology |
| 61 | Herrmann et al., 2014 | Rotator cuff | Literature Review | 5 | Developed an algorithm for treating rotator cuff pathology. |
| 62 | Hopman et al., 2015 | Rotator cuff | Literature Review | 5 | Established clinical practice guidelines including three algorithms and 35 recommendations for treating rotator cuff pathology in the workplace. |
| 63 | Hsu & Keener, 2015 | Rotator cuff | Literature Review | 5 | Developed an algorithm for treating rotator cuff pathology. |
| 64 | Iannotti & Kwon, 2005 | Rotator cuff | Literature Review | 5 | Developed two algorithms for treating rotator cuff pathology (shoulder pain and non-operative management). |
| 65 | Ladermann et al., 2015 | Rotator cuff | Literature Review | 5 | Developed an algorithm for treating massive rotator cuff tears. |
| 66 | Nam et al., 2012 | Rotator cuff | Literature Review | 5 | Developed an algorithm for the treatment of a massive, irreparable rotator cuff tear. |
| 67 | Nazarian et al., 2013 | Rotator cuff | Literature Review | 5 | Developed three imaging algorithms for evaluating suspected rotator cuff pathology. |
| 68 | Okoro et al., 2009 | Corocoid impingement | Literature Review | 5 | Developed an algorithm for the diagnosis and management of coracoid impingement. |
| 69 | Pedowitz et al., 2010 | Rotator cuff | Systematic review/consensus guidelines | 2a | Established clinical practice guidelines (31 recommendations) for the management of rotator cuff pathology. |
| 70 | Thes et al., 2015 | Rotator cuff | Literature Review | 5 | Developed an algorithm for treating massive rotator cuff tears. |
| 71 | Wolf et al., 2007 | Rotator cuff | Literature Review | 5 | Developed an algorithm for treating rotator cuff pathology in patients older than 50 years of age. |
| 72 | Cadogan et al., 2016 | Subacromial pain | Literature Review | 5 | Developed an algorithm for identifying patients with subacromial pain. |
| 73 | Diercks et al., 2014 | Subacromial pain | Systematic review/consensus guidelines | 2a | Established clinical practice guidelines for the diagnosis and treatment of subacromial pain. |
| 74 | Amini et al., 2018 | Undifferentiated pain – traumatic injury | Systematic review/consensus guidelines | 2a | Developed appropriate use criteria for imaging. |
| 75 | Andrews, 2005 | Undifferentiated pain | Literature Review | 5 | Developed a nonoperative treatment algorithm for chronic shgmoulder pain defined as pain lasting longer than 6 months. |
| 76 | Arce et al., 2013 | Undifferentiated pain | Literature review/consensus guidelines | 5 | Developed consensus guideline for several shoulder key topics including rotator cuff pathology, shoulder instability, and ACJ disorders. |
| 77 | Artus et al., 2014 | Undifferentiated pain | Literature review/consensus guidelines | 5 | Developed an algorithm for the diagnosis and treatment of undifferentiated shoulder pain. |
| 78 | Caroit et al., 1997 | Undifferentiated pain | Literature review/consensus guidelines | 5 | Presented recommendations from a consensus conference regarding imaging for the undifferentiated, unoperated shoulder. |
| 79 | Codsi, 2007 | Undifferentiated pain | Literature Review | 5 | Developed an algorithm for the diagnosis and treatment of undifferentiated shoulder pain. |
| 80 | Donovan & Paulos, 1995 | Undifferentiated pain | Literature Review | 5 | Developed an algorithm for the diagnosis and treatment of undifferentiated shoulder pain. |
| 81 | Murphy & Carr, 2009 | Undifferentiated pain | Literature Review | 5 | Developed an algorithm for the management of patients presenting with undifferentiated shoulder in general practice including an algorithm for identifying red flags. |
| 82 | O’Kane & Toresdahl, 2014 | Undifferentiated pain – traumatic injury | Meta-analysis | 2a | Developed an algorithm for the evaluation of shoulder pain resulting from a traumatic injury. |
| 83 | Robb et al., 2009a | Undifferentiated pain | Systematic review/consensus guidelines | 2a | Established clinical practice guidelines for the diagnosis and treatment of shoulder injuries. |
| 84 | Robb et al., 2009b | Undifferentiated pain | Systematic review/consensus guidelines | 2a | Established clinical practice guidelines for the diagnosis and treatment of shoulder injuries. |
| 85 | Small et al., 2018 | Undifferentiated pain – atraumatic injury | Systematic review/consensus guidelines | 2a | Developed appropriate use criteria for imaging. |
| 86 | Uhthoff & Sarkar, 1990 | Undifferentiated pain | Literature Review | 5 | Developed an algorithm for managing shoulder pain caused by soft-tissue disorders. |
| 87 | Varela et al., 2013 | Undifferentiated pain | Literature Review | 5 | Developed an algorithm for the diagnosis and treatment of undifferentiated shoulder pain. |
| 88 | Wise et al., 2011 | Undifferentiated pain – acute pain less than 2 weeks | Systematic review/consensus guidelines | 2a | Developed appropriate use criteria for imaging. |
